# Critical Illness Polyneuropathy in a Child: A Case Report

**DOI:** 10.7759/cureus.56703

**Published:** 2024-03-22

**Authors:** Shiji Chalipat, Jyothsna Sree Madala, Sanjay Chavan, Sudhir Malwade, Shilpa Baviskar

**Affiliations:** 1 Pediatric Neurology, Dr. D. Y. Patil Medical College, Hospital and Research Centre, Dr. D. Y. Patil Vidyapeeth (Deemed to Be University), Pune, IND; 2 Pediatrics, Dr. D. Y. Patil Medical College, Hospital and Research Centre, Dr. D. Y. Patil Vidyapeeth (Deemed to Be University), Pune, IND

**Keywords:** septicemia, meningitis, intensive care, myopathy, critical illness polyneuropathy

## Abstract

Critical illness polyneuropathy (CIP) and myopathy (CIM) are underreported conditions in critically ill children with prolonged intensive care unit stays and mechanical ventilation. We report a case of a 10-year-old boy with pneumococcal meningoencephalitis with severe sepsis and multiorgan dysfunction. The child required prolonged ventilation, sedation, and inotropic support. He had repeated extubation failures and the development of quadriparesis with areflexia. Electrophysiology studies were consistent with CIP with acute motor and sensory axonal polyneuropathy and elevated muscle enzymes. He was treated with supportive measures and physiotherapy along with management of the underlying condition. He recovered slowly over 68 days with a good recovery with a modified Rankin’s scale score of 4 on discharge. There is a need to pay attention to all critically ill children and should have a high index of suspicion for the development of CIP/CIM which can have an impact on course and outcome.

## Introduction

Critical illness polyneuropathy (CIP) and myopathy (CIM) or intensive care unit-acquired weakness (ICU-AW) have been a significant cause of neuromuscular weakness in critically ill children with prolonged intensive care stays. The earliest description of this condition was done by Osler who described it as ‘rapid loss of flesh’ in patients with severe sepsis [1]. Bolton et al. described CIP as acute flaccid quadriparesis with repeated extubation failures from a ventilator [2]. The exact pathogenesis of CIP is unknown and the presumed mechanism is disturbance of microcirculation causing tissue hypoxia and endoneural edema causing primary axonal degeneration and myoneural necrosis [3]. Other proposed mechanisms are an acquired sodium channelopathy and mitochondrial impairment with oxidative stress [4,5].

Here we describe a child with pneumococcal meningoencephalitis, severe sepsis, and multiorgan dysfunction, who developed CIP during an intensive care stay.

## Case presentation

A 10-year-old healthy boy was admitted with a two-day history of fever, headache, and vomiting in status epilepticus requiring multiple antiseizure medications. He was ventilated in due course of management and was commenced on empirical intravenous antibiotics, acyclovir, and intravenous fluids.

Details of blood investigation parameters and their trends over time are depicted in Table 1.

**Table 1:** Trend of blood parameters as per day of admission Hb - Hemoglobin, TLC - Total leukocyte count, ALT - Alanine aminotransferase, AST - Aspartate aminotransferase, ALP - Alkaline phosphatase, PT - Prothrombin time, INR - International Normalized Ratio, APTT - Activated partial thromboplastin time

Investigations	Normal Values	Day 1	Day 5	Day 10	Day 35
Hb (g/dl)	10.5-18	11.9	10.4	10.9	9.2
TLC (/mm^3^)	4000-12000	12100	7200	18800	8200
Platelets (/µl)	150,000-400,000	1.39lakhs	94,000	63,000	236000
C-reactive protein (mg/dl)	0.06-0.79	273	178	156	8
Total bilirubin (mg/dl)	<1	0.64	0.3	0.5	0.12
AST (U/L)	10-40	17	82	230	39
ALT (U/L)	5-45	28	76	140	100
ALP (U/L)	140-560	101	103	101	103
Total protein (g/dl)	6.4-8.1	5.6	4.6	4.8	5
Serum Albumin (g/dl)	3.5-5.6	3.6	2.4	2.6	4.3
Serum Sodium (mmol/L)	134-143	136	158	146	132
Serum Potassium (mmol/L)	3.3-4.6	3.1	3.9	4.6	4.4
Serum Chloride (mmol/L)	98-106	107	120	104	100
Serum Calcium (mg/dl)	8.4-10.2	7.4	7.2	7.1	9.3
Serum Phosphorus (mg/dl)	3.7-5.6	3.1	3.0	2.9	4.4
Serum Magnesium (mg/dl)	1.5-2.3	2.1	2.13	2.23	1.79
Ionic Calcium (mmol/L)	1.12-1.23	1.11	1.18	1.2	1.34
Urea (mg/dl)	7-18	18	22	20	17
Creatinine (mg/dl)	0.33-0.88	0.67	0.7	0.5	0.37
PT/INR (sec)	12.1-14.6/0.98-1.20	17.9/1.45	21.3/1.7	12.3/0.98	11.2/0.96
APTT (sec)	28-45.0	33.1	32	21.8	30.3
D-dimer (ng/ml)	400-2270	4870	4562	4246	430
Ferritin (ng/ml)	10-300	474.89	467	412	313

Cerebrospinal fluid (CSF) analysis was abnormal and the latex agglutination test was positive for *Streptococcus pneumoniae*. The detailed CSF analysis is depicted in Table 2. The corresponding blood sugar level during the CSF study was 98 mg/dL (normal level: 90-140 mg/dL). CSF polymerase chain reaction (PCR) tests for neurotropic viruses were negative as shown in Table 3.

**Table 2:** Details of cerebrospinal fluid analysis CSF - cerebrospinal fluid, CBNAAT - Cartridge-based nucleic acid amplification testing

CSF Analysis	Observed Value	Normal Value
Total cells	900	<5 cells
Polymorphs	80%	0
Lymphocytes	20%	<5
Glucose	22 mg/dl	45-80 mg/dl
Protein	22 mg/dl	20-40 mg/dl
Latex agglutination test	Positive - *Streptococcus pneumoniae*	Negative
CBNAAT for *Mycobacterium tuberculosis*	Negative	Negative

**Table 3:** CSF PCR test result for neurotropic viruses CSF - Cerebrospinal fluid, PCR - Polymerase chain reaction

Neurotropic Viruses	CSF PCR Results
Japanese encephalitis virus	Negative
Herpes simplex virus	Negative
Enterovirus	Negative
Adenovirus	Negative

**Figure 1:**
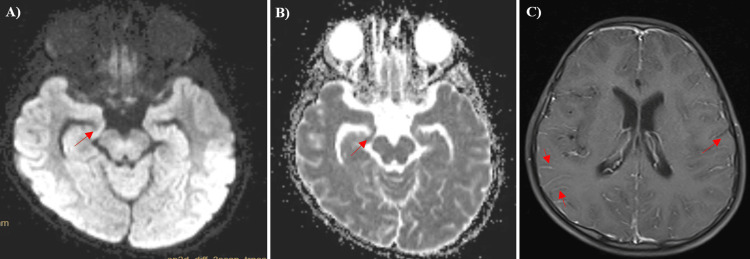
Images A and B - MRI brain showing diffusion restriction (arrow) in DWI (diffusion-weighted image) and ADC (apparent diffusion coefficient) sequences in the bilateral hippocampal area; Image C - MRI brain T1 sequence contrast image - showing leptomeningeal enhancement (arrows).

Over the next 72 hours, the child deteriorated having persistent fever and multisystem involvement in the form of raised liver enzymes, persistent hypotension, fall in platelet count, and decreased cardiac contractility with ejection fraction. MRI brain showed evidence of leptomeningeal enhancement suggestive of meningoencephalitis and diffusion restriction in the right hippocampus secondary to status epilepticus. MRI brain changes have been demonstrated in Figure 1.

**Figure 2:**
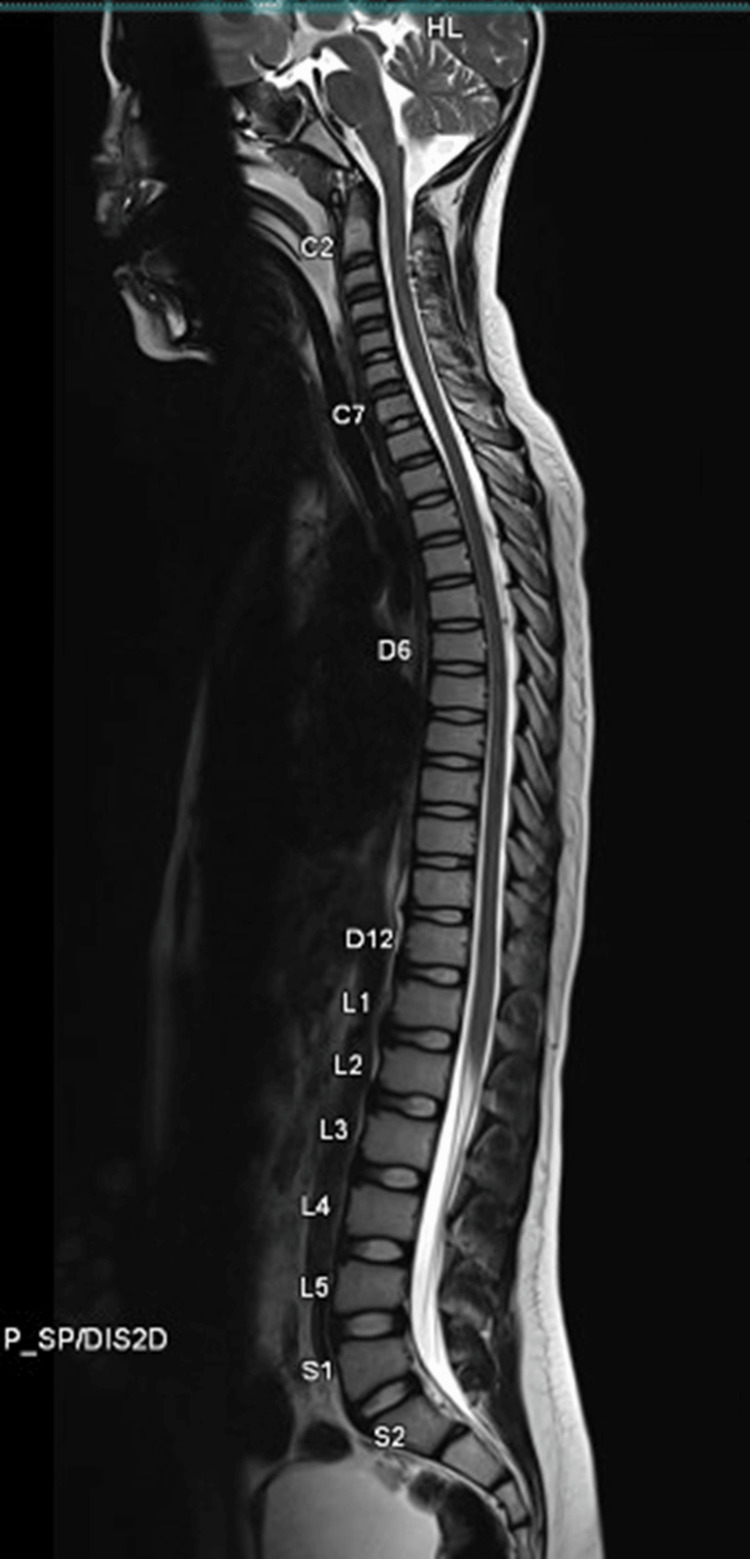
MRI whole spine screening showed no abnormal findings

On the 10th day of admission, when the child gradually recovered and sedation was weaned off before extubation, we noticed quadriparesis, generalized hypotonia with areflexia, and absent plantar response. Bladder involvement could not be assessed as the child was already catheterized. Normal MRI spine ruled out spinal cord pathologies. MRI's whole spine has been depicted in Figure 2.

The serum creatine phosphokinase (CPK) enzyme level was 532 U/L (80-120 U/L). Nerve conduction study (NCS) findings were suggestive of mixed sensory-motor axonal polyneuropathy. The motor conduction values of NCS are shown in Table 4 and sensory conduction values of NCS are shown in Table 5.

**Table 4:** Nerve conduction study - motor conduction study values CMAP - Compound Muscle Action Potential; mv - Millivolt; µV - Microvolt; msec - milliseconds; m/s - meter/second

Motor Nerves	CMAP Amplitude (mV)	Normal Value (mV)	Distal Latency (msec)	Normal Value (msec)	Conduction Velocity (m/s)	Normal Value (m/s)
Right Median Nerve	6.3 mV	10.9±2.7 mV	2.4 msec	3.3±0.4 msec	53.7 m/s	58±4 m/s
Left Median Nerve	6.6 mV	10.9±2.7 mV	2.9 msec	3.3±0.4 msec	52.2 m/s	58±4 m/s
Right Ulnar Nerve	5.1 mV	10.7±2.4 mV	2.6 msec	2.5±0.3 msec	54 m/s	62±5 m/s
Left Ulnar Nerve	6.4mV	10.7±2.4 mV	2.9 msec	2.5±0.3 msec	61.5 m/s	62±5 m/s
Right Peroneal Nerve	Absent response	5.4±2.0 mV	Absent response	4.2±0.7 msec	Absent response	51±5 m/s
Left Peroneal Nerve	0.6 mV	5.4±2.0 mV	4.7 msec	4.2±0.7 msec	44.1 m/s	51±5 m/s
Right Tibial Nerve	3.2 mV	11.8±3.6 mV	2.6 msec	4.0±0.7 msec	45.9 m/s	50±4 m/s
Left Tibial Nerve	3.4 mV	11.8±3.6 mV	4.8 msec	4.0±0.7 msec	43.2 m/s	50±4 m/s

**Table 5:** Nerve conduction study - sensory conduction study values SNAP - Sensory Nerve Action Potential; µV - microvolt; msec - milliseconds; m/s - meter/second

Sensory Nerves	SNAP Amplitude (µV)	Normal Value	Distal Latency (msec)	Normal Value	Conduction Velocity (m/s)	Normal Value
Right Median Nerve	18.2 µV	50±15 µV	2.8 msec	2.9±0.3 msec	61.5 m/s	66±4 m/s
Left Median Nerve	17.4 µV	50±15 µV	2.72 msec	2.9±0.3 msec	60 m/s	66±4 m/s
Right Ulnar Nerve	15.8 µV	41±12 µV	2.4 msec	2.6±0.3 msec	61 m/s	67±5 m/s
Left Ulnar Nerve	16.2 µV	41±12 µV	2.3 msec	2.6±0.3 msec	59 m/s	67±5 m/s
Right Sural Nerve	3.6 µV	18±8 µV	3.4 msec	3.6±0.3 msec	69 m/s	52±6 m/s
Left Sural Nerve	4.2 µV	18±8 µV	3.3 msec	3.6±0.3 msec	52.2 m/s	52±6 m/s

**Figure 3:**
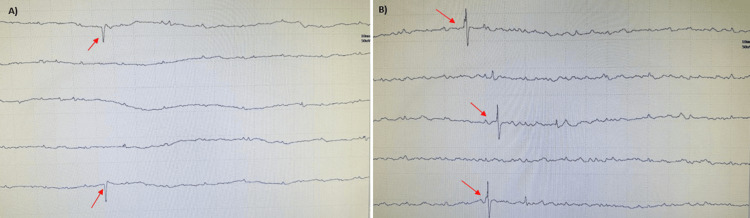
Electromyography study showing abnormal spontaneous activity - (A) positive sharp waves (arrows); (B) fibrillation potentials (arrows)

A limited electromyography (EMG) study was done in the right vastus medialis and tibialis anterior in the right lower limb. It showed normal insertional activity, abnormal spontaneous activity like fibrillation potentials, and positive sharp waves which have been demonstrated in Figure 3. Motor Unit Action Potentials (MUAPs) could not be assessed. Electrophysiological studies were in accordance with CIP/CIM. Muscle biopsy, which is considered a gold standard investigation to differentiate CIP and CIM, could not be done.

Rehabilitation was initiated with active and assistive exercises. A tracheostomy was needed and supportive management continued. After multiple extubation failures, the child was extubated successfully after 28 days and eventually shifted out of the pediatric intensive care unit (PICU) after 42 days. He was discharged after 68 days of hospital stay and was able to walk with support (Modified Rankin Score (MRC) - 4). After one month of follow-up, the child was able to walk without support (MRC-3) with improvement in muscle tone and bulk and NCS showed improvement in Compound Muscle Action Potential (CMAP) amplitude in motor nerves with normal sensory NCS.

## Discussion

CIP/CIM is an important cause of neuromuscular weakness seen in children who are critically ill and require prolonged ventilator care in the PICU. The exact incidence of CIP/CIM in children is not known. The first largest prospective study in children by Ban Well et al. showed an incidence of 1.7% [6]. Another prospective study in children from North India reported less than 4.7% incidence (95% CI 0-4.75) [7]. These values are much lower than in adults [8], maybe because of poor recognition of this condition in children [9].

The most common risk factors for CIP/CIM are severe sepsis, systemic inflammatory response syndrome (SIRS), multiorgan dysfunction syndrome (MODS), and mechanical ventilation for more than seven days [10]. Other independent risk factors reported are female gender, hyperglycemia, hypoalbuminemia, dyselectrolytemia, thrombocytopenia, elevated liver enzymes, prolonged sedation, neuromuscular blocking agents, corticosteroids, and inotropes [11].

Our case presented pneumococcal meningoencephalitis complicated with severe sepsis, SIRS, and cardiac dysfunction requiring intensive care and prolonged ventilation. He also had hypoalbuminemia, elevated liver enzymes, thrombocytopenia, prolonged prothrombin time, and had received steroids and inotropes. Like other cases of CIP/CIM, our case also presented with acute quadriparesis, muscle atrophy, areflexia or hyporeflexia, and difficulty in weaning off from the ventilator. Electrophysiological studies favored CIP as per Bolton’s diagnostic criteria [11]. The multicentric CRIMYNE (CRitical Illness MYopathy and/or Neuropathy) study [12] as well as Kasinathan et al [7] has reported the significance of common peroneal nerve study and the findings in CIP. A reduction of 25% in CMAP amplitude had 100% sensitivity and 67% specificity for the detection of CIP. In our case, there was an absent response in the right peroneal nerve and a significant decrease in CMAP amplitude in the left peroneal nerve. In the study by Kasinathan et al., they attempted the simplified serial isolated common peroneal testing as a practical alternative to the clinical methods to detect CIP [7].

A multidisciplinary approach is required for overall success in the management of these patients. There are no proven therapies that prevent or reverse CIP/CIM. Several therapies suggested for prevention include proper nutritional intervention, antioxidants, and hormonal therapies without any definite evidence [13]. There is substantial evidence for insulin therapy to keep strict glycemic control, which might decrease the incidence of CIM/CIP and the need for prolonged ventilation [14].

CIP/CIM has a great impact on the outcome of the patients. It leads to the prolonged need for ventilator support because of phrenic nerve and diaphragmatic muscle involvement, increased hospital stay, and morbidity and mortality [13]. The prognosis depends on the extent of nerve and muscle damage. Full recovery often occurs in mild to moderate cases within weeks, whereas improvement takes months in severe cases with higher mortality [13]. Our patient had a protracted stay of nearly two months in the hospital.

## Conclusions

CIP/CIM alone or in combination is relatively rare and an under-reported condition in the pediatric population. It has a multifactorial etiology and is diagnosed by clinical examination along with electrophysiological studies. CIP is associated with great implications in the course of illness, length of intensive care stay, and outcome in critically ill children. Large prospective studies are needed to better characterize the incidence and evolution of CIP/CIM in critically ill children.
